# Cross-over analysis of the climate-change delta situation of the cities Gdansk (Baltic-sea) and Rotterdam (Nord-sea)

**DOI:** 10.12688/openreseurope.13125.1

**Published:** 2021-03-24

**Authors:** Fred Sanders, Hugo Sanders, Karen Jonkers

**Affiliations:** 1 CPONH NGO, Heerhugowaard, The Netherlands; 2 Urbanism Department, Delft University of Technology, Delft, The Netherlands

**Keywords:** climate change, mitigation, adaptation, built environment, coastal cities

## Abstract

Gdansk and the city Haarlem in the Netherlands share a long-term relationship that started with the establishment of Dutch Mennonites in the Vistula delta in the 16th Century. A small city was founded called Holland and these immigrants reclaimed the surrounding delta area. This area of 1,000 km ^2^, with hundreds of small ‘polders’ separated and defended by 17,000 dikes, has become an important agricultural area for the whole of Poland, similar to the Rhine delta in the Netherlands. Despite these civil defense works in the past, both coastlines nevertheless experienced floods: the Dutch southwest coast in 1953, Dutch Rhine riverbank in 1993 and 1995, and Vistula delta recently in 2001. Climate change figures show that both the Polish Gdansk and Dutch Rhine deltas will suffer flooding with sea level rises, with accumulating severe rainfall accompanied by high river levels. Although both the Baltic Sea and the North Sea are next to each other and coupled to the Atlantic Ocean, there are differences in how soon or severely climate change trends, such as seawater level rises and water thrust, become critical. From cross-over analysis it can be concluded that Poland and the Netherlands have a virtually identical approach when it comes to climate change impacts on their current situation. With regard to long-term climate change, the Netherlands is exploring the future in a planned manner with the development of new scenarios for the protection of cities. The enclosure of the Baltic Sea, on the other hand, probably offers more options for exchanging knowledge with neighbor states. In that respect, the Netherlands is more isolated in their situation with the North Sea and its Delta Plan. The situation of Gdansk and Rotterdam is quite similar; these cities can take steps forward by learning from each other’s actions.

## The Gdansk Baltic-sea climate-change delta situation

The Baltic Sea borders nine countries, is 1,600 km long, 193 km wide at its maximum and only 55 meters deep on average. The climate differences are huge in this area; strong long winters in the north and a mild continental climate in the south. This affects the water conditions and therewith the coastal circumstances and its water-related threats. Climate change influences this situation due to sea level rises, winter sea level changes, changing pole-tides, wind-induced water backlash, and the increasing water level changes in the sea joining rivers ( Ekman, 2009). The interaction of these influencing factors is complex, and the water level and the fluctuations therein are particularly location dependent ( Omstedt *et al.*, 2004). These factors have changed more drastically over recent years due to climate-change ( BACC Author Team, 2008).

**Figure 1.**
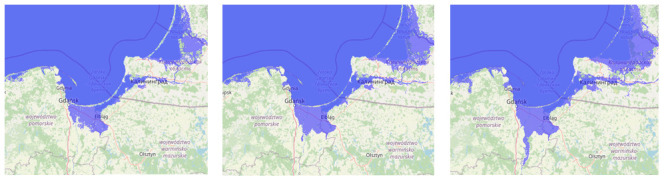
The land flooding at 1-, 5- and 10-meters sea level rise from left to right (pictures made by using www.floodmap.net, an open-access governmental website, with OpenStreetMap © OpenStreetMap contributors).

The situation of the Scandinavian land north of the Baltic Sea is quite extraordinary. Here the land still rises every year after the former ice ages. The weight of the melted ice sheets is taken away and the land is returning to its former position. This is quite different from the southern Baltic Sea shore where the land is subject to subsidence ( Haerff *et al.*, 2017). There are many developing factors that together predict that the coasts around the Baltic Sea will change remarkably due to climate change in the years to come ( Labuz, 2015). According to the EC Inventory, the Polish coastal zone is highly vulnerable to climate change, although relatively few people live along this 634 km coastline (see Figure 1). 

**Figure 2.**
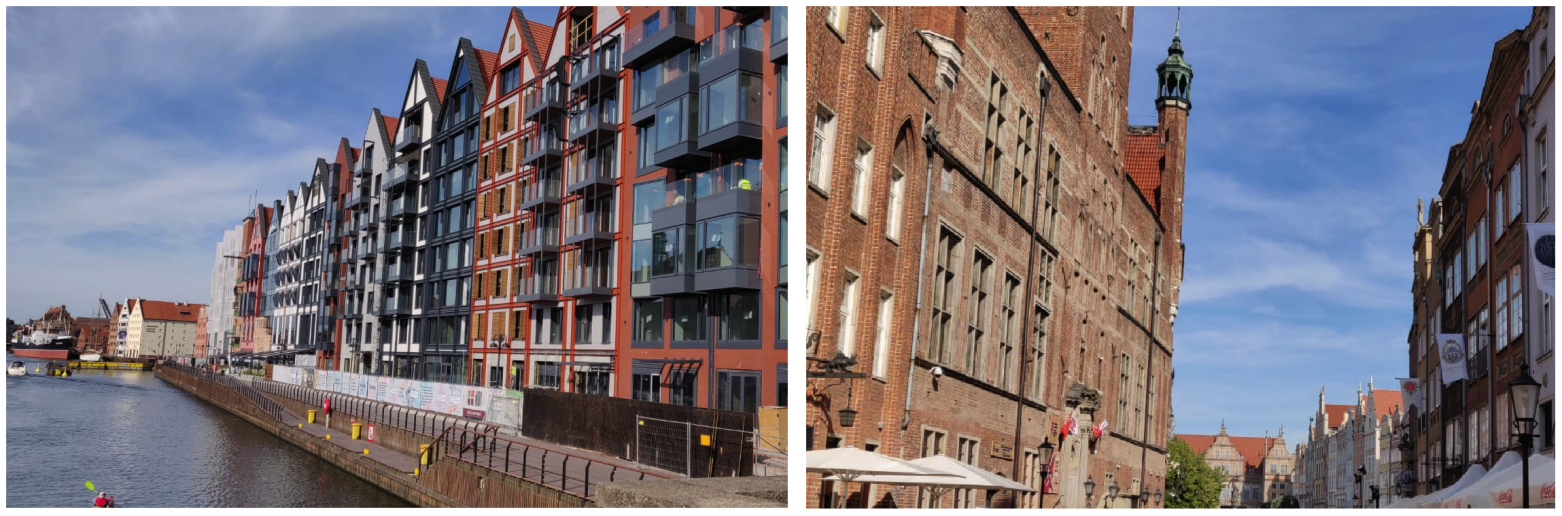
Impression of Gdansk city and waterfront (pictures by H.H.C Sanders, one of the authors).

Gdansk is situated along the coast in the Gulf of Gdansk, west of the great Zulawy polder area, also called the Vistula delta because of the river Vistula that dominates the area. The area is special because the Vistula delta is a nature reserve, is responsible for 6% of the Polish agricultural food production and is an important source of drinking water. Handling the climate change water-related impact on this delta is especially urgent because of the increasing accumulation of high river water levels and the impact of severe rainfall in the last decade, especially coming from the higher hinterland. For the city of Gdansk, the situation has become more urgent in recent years as the city border lies at a river waterfront open to the Baltic Sea, see Figure 2.

The area of the city of Gdansk is characterised by an anthropogenic landscape of polders, dried marshlands and former oxbow lakes that have suffered periodic flooding during the last few centuries. The situation changed with the construction of the bypass channel of the Vistula river east of Gdansk city in 1895, leading directly to the Gulf of Gdansk ( WMO, 2005). However, climate change caused new flooding by cumulating sources in 1924 (huge snowmelt and rainfall), 1955 (strong storm), 1956 (ice-jam), 1983 (accident), and 2001 (torrential rainfall) ( Cyberski *et al.*, 2006). The coastal situation of Gdansk situated within the Gulf of Gdansk is thus determined by several climate change factors whereby the configuration of the Baltic Sea as a whole plays a role. The main influencing factors are: the higher seawater temperature; decreasing ice formation; increasing water import from rivers with their fluctuations along the Baltic Sea coast and in specific locations; increasing water backlog due to storm surges and more heavy clustered rainfall; the rising and falling of the land, which differs from the southern coast on the northern coast, whereby the effects differ locally along the 1,900 km long coast ( Ekman, 2009). Although the first computer simulations are under development (at institutes in Poland and at NIOZ in the Netherlands), the interaction of these factors is difficult to predict ( https://www.climatechangepost.com/poland/climate-change/).

The current situation is that Gdansk city and the eastern polder area remains dry by constant action: dike reinforcement and drainage pumping the water out of the delta by channels and the rivers to the sea. For the city of Gdansk, the planned climate change measures are: 1) long term coastal protection strategies; 2) flood warning system for the safety of residents; 3) canaling of the Vistula river to the Gulf of Gdansk in 1840 and 1895; 4) reservoirs for flood protection built on streams in cascades; 5) expansion of the city drainage system for capturing heavy rainfall; 6) education and support for rain gardens to residents ( ModE, 2010; ModE, 2019).

Besides the situation in Gdansk and its surroundings, the question is: can the water-related approaches to the situation be seen as independent from the impact of climate change in the other corners of the Baltic Sea? For instance; the reduction in ice-formation in the northern part of the Baltic Sea will speed up the rise in seawater level on the southern coast as well ( Ekman, 2009). Although the costs of extra defensive measures at this south coast will be less than the loss of value in the coastal area of Poland, the total investment will soon be too much for the region ( Zeidler, 2015). The insights and experiences of other countries located around the Baltic Sea could be helpful for this, to copy successful measures or to cooperate with on this challenge of climate change.

Another, potentially faster, approach to determine the effect on the Polish coast and in particular on the largest city of Gdansk is to study the most critical factors and lessons learned about the Baltic Sea. This could be achieved through: 1) learning approaches from the highly similar situations of the Baltic states Estonia, Latvia and Lithuania; 2) studying the relationship of land-rise and the Baltic Sea, for instance the situation of nearby Finland; and 3) learning from the measures taken in Stockholm to safeguard the old town and the capital invested. Additionally, influences of climate change on nature and its biodiversity should get attention, because deforestation and destruction of river-vegetation accelerates the discharge of water from rivers; 4) the nearby situation of the Baltic States and Finland could be helpful in clarifying this influence besides conducting local studies.

## The Rotterdam North Sea climate change delta situation

**Figure 3.**
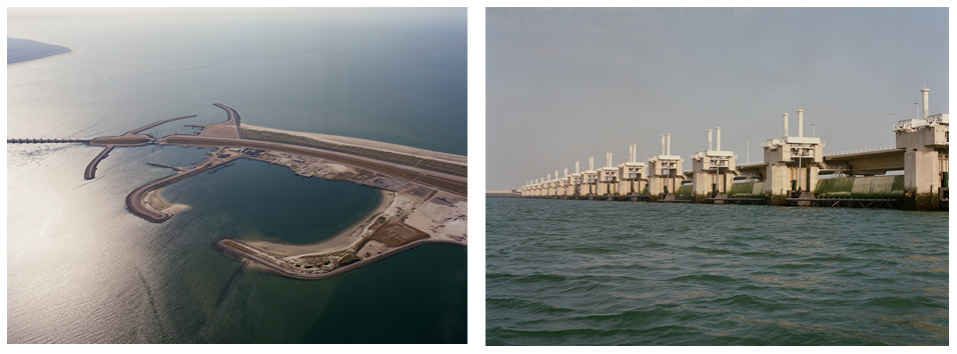
Impression of the “Oosterschelde’ dam (left), and construction (right) ( https://beeldbank.rws.nl, Rijkswaterstaat /Jaap Boelens (left) and Henri Cormont (right)).

**Figure 4.**
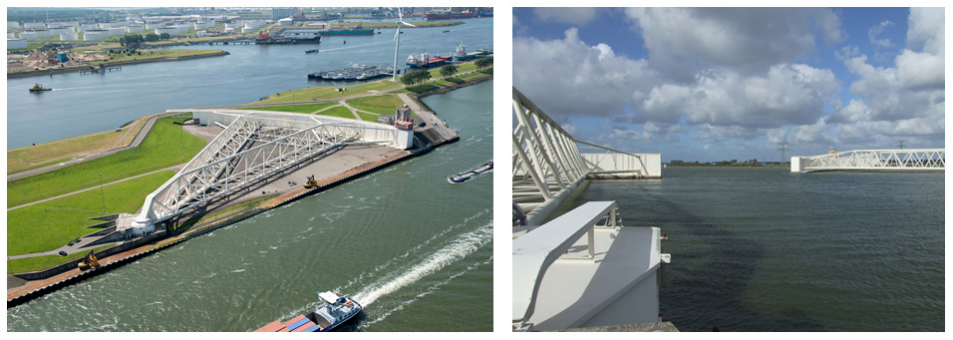
Maeslant-barrier, Rotterdam ( https://beeldbank.rws.nl, Rijkswaterstaat/JoopvanHoudt).

The last severe flood in the Netherlands, called ‘Watersnoodramp’, happened in February 1953 whereby 165 hectares of land flooded, mainly in the southwest of the country. In total, 1836 people lost their lives, 10k people lost their homes from the 72k that had to be evacuated, and approximately 50k cows and 150k chickens died. This unexpected disaster became the start of ‘Deltaplan’ to protect the Netherlands from the sea in the future. With the installation of the governmental ‘Delta Commission’ in 1953 two months later, plans to shorten the coast with 700 km of dikes started ( Deltawet, 1958), see Figure 3. During the years after, the safety of the important city of Rotterdam was ensured by this program through construction of the ‘Maesland barrier’, the most Northern dam shown in the map in Figure 4.

In 2010 the construction ended unofficially; due to climate change, the Delta Commission was directed to make plans to address seawater level rises and other climate change coupled impacts in the future ( Deltawet, 2011). The most recent advice from this commission of 15 September 2020 ( Deltaprogramma, 2021) focussed on: 1) water security, 2) freshwater availability; and 3) spatial design, all related to climate change impact. The Delta Commission thereby concluded that the Dutch coastal defence system can handle the 100 cm sea level rise that may happen at the earliest in 2100 according to worse-case scenarios based on IPCC institute data. However, in case of a speeding up of sea level rise, some of the new coastal dikes have to be reinforced, especially the recent Maesland barrier, which offers safety for the important harbour of Rotterdam, see Figure 4.

**Figure 5.**
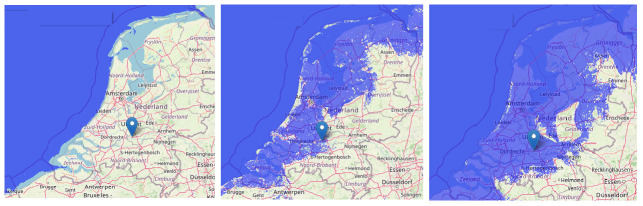
The situation of the Netherlands in relation to sea level: actual situation behind the dikes (left), flooding in case of 1.0 meter higher sea level without dikes (middle), and in case of 10.0 meter (right) (pictures made by using www.floodmap.net, an open-access governmental website, with OpenStreetMap © OpenStreetMap contributors).

Based on the recent Delta-Commission statement, the Dutch coast system can handle 10 meters of sea level rise totally, assuming there are no budget constraints. Flood scenarios show that 50% of the country will be flooded in that extreme situation ( Haasnoot *et al.*, 2020), which concerns the part of the country where almost 80% of the country population lives and where the cities Amsterdam and Rotterdam, the most important economic motors of the country, are situated, see Figure 5.

More worrisome with the rise in sea level is the major operation to gradually strengthen and raise all dikes, the primary sea defence and the polder dikes, and the increasing risk due to the effects of climate change to the primary sea defence. The most drastic factors behind this risk to the primary sea defence are higher temporary river levels, drier periods and more intense and prolonged periods of rainfall. These effects have an impact on the overload of sewer systems in cities, the subsidence of old houses, and the fertility of agricultural land for which salinization is an important factor. The production of Dutch tulip bulbs and potatoes already suffers easily from low salinization. The most drastic impacts, however, is the expected future flooding of the large cities in the western part of the Netherlands and the stagnation of economic growth in the adjacent industrial sites, which will then have a major impact on a national scale.

**Figure 6.**
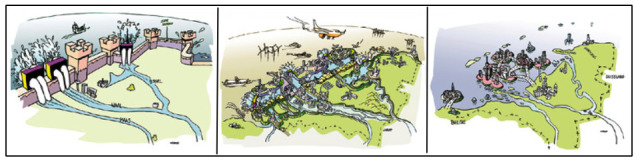
Three scenarios for handling extreme sea level rise in the Netherlands; the three scenarios from left to right. (Copied from the Hackathon report with permission of Deltares) ( Haasnoot *et al.*, 2019).

To stay ahead of this development, more drastic measures in the coming years will be needed according to the Delta Commissioner, advising his institute in 2019 on how to address this impact of water-related climate change. In 2017 a ‘Policy Hackathon’ was organized ( Haasnoot *et al.*, 2019) to explore robust scenarios and solutions for the long run, resulting in the three main scenarios for handling extreme sea level rise in the Netherlands: 1. defending the delta by fortification using a high dike around the country; 2. defending the delta by creating a new barrier off the coast with the advantage of new land for housing and tourism; 3. defending the most important economic areas including the cities using dikes with the sea coming into the land, see Figure 6.

These extreme scenarios come from the conclusion that in the case of sea level rises of more than 10 meters, the existing system of polders with primary and secondary dikes will collapse because the liveability due to salination, lack of fresh drinking water and impaired mobility at the end cannot be sustained. An old, far more drastic proposition was brought into this discussion recently; to close the North Sea from the ocean with dikes. The proposal has many disadvantages, especially for biodiversity as the area transitions from salt to freshwater at the end, but it would also lower many costs. It shows how the Dutch society is looking forward, no option being excluded. It also confirms the need for choices to be made this century.

## Cross-over analysis and conclusions

When comparing the climate-change adaptive measures of the cities Gdansk in Poland and Rotterdam in the Netherlands, both are situated in the heart of the country’s most important delta and both located are similarly in relation to the sea and to the hinterland. Both cities: 1) are located by an enclosed sea in the northern hemisphere, in a delta with surrounding ‘polder’ areas; 2) are located in an area in which the land to the north is rising and the land is subsiding in the south; 3) face sea level rise; 4) experience backwater during storms; and 5) are located on a river in the delta where the river level fluctuates strongly, caused by meltwater upstream, heavy rainfall alternated with dry periods, with peaks occurring when these influences coincide. The situations are so similar that it becomes interesting to learn from each other's experiences in the field of climate change interventions. 

**Figure 7.**
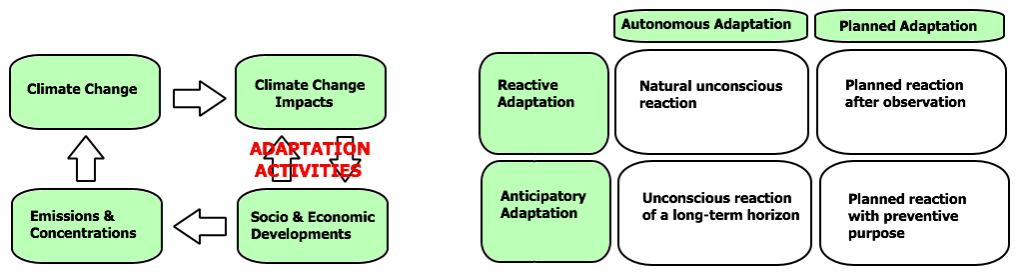
Diagram for climate-change adaptive relations (left) and adaptive measures (right) (Adapted from IPCC 2007 Report, with permission of Cambridge University Press, 20 January 2021) ( IPCC, 2007).

Therefore, the ‘Astra’ model (based on IPCC models) that categorizes adaptive measures is used for comparison as a base for analyses ( Hilpert *et al.*, 2007). This model divides actions into ‘Autonomous’ and ‘Planned’ adaptation activities and divides these into ‘Reactive and ‘Anticipatory’ measures, see Figure 7.

The different choices made for Gdansk and Rotterdam are interesting, and these can be of interest to the other city. The cross-over analysis with a focus on ‘Planned Adaptation’ can be seen in Table 1.

**Table 1.** Inventory of the Gdansk and Rotterdam ‘Adaptive measures’ related to climate change.

Adaptive Activities	Gdansk Planned Adaptation	Rotterdam Planned Adaptation
Reactive Adaptation	• Plan coastal protection strategy • Flood warning system residents. • The ‘Vistula’ canals to ‘Gdansk Gulf’ • Reservoirs for flood in rivers • City drainage system for heavy rainfall	• New ‘1985 Deltaplan’ with update 2015 • The ‘Maesland’ barrier • Plan ‘Space adaptation’ • Widening upstream river beds. • City reservoirs for heavy rainfall.
Anticipatory Adaptation	• New riverfront architecture	• New ‘Deltaplan’ scenario development

The situations of Gdansk and Rotterdam are quite similar; these cities can take steps forward by learning from each other’s actions. Both cities and their countries are aware of their vulnerability to climate-change impacts because of their delta situated locations. Secondly, the situation in their deltas are quite similar, and they both have actual action plans to deal with climate change impacts. The difference for Gdansk is that the hinterland behind the city rises so that rainwater flows into the city faster. That is why Gdansk provides storage reservoirs in the city and an extensive sewerage system, more than Rotterdam. Due to the vulnerability of the coastal situation, Gdansk is looking towards a solution involving a warning system and movable barriers that has been built in the Netherlands. The differences may in approach be due to the different financial situations of both countries. The approaches of these cities to highwater levels in the rivers passing by appear to be the same, although the technical interpretations are different because of the landscape differences.

With regard to long-term climate change, the Netherlands is exploring the future in a planned manner with the development of new scenarios for the protection of cities, where Poland is entering a phase where more urban development and architectural solutions are sought for their coastal cities. The enclosure of the Baltic Sea, on the other hand, probably offers more options for exchanging knowledge with neighbor states. In that respect, the Netherlands is more isolated in their situation with the North Sea and their Delta Project.

## Data availability

All data underlying the results are available as part of the article and no additional source data are required.
