# Crossover comparison of climate-change adaptation measures taken in the Gdansk (Baltic-sea) and Rotterdam (Nord-sea) deltas.

**DOI:** 10.12688/openreseurope.13125.2

**Published:** 2021-10-13

**Authors:** Fred Sanders, Hugo Sanders, Karen Jonkers

**Affiliations:** 1 CPONH NGO, Heerhugowaard, The Netherlands; 2 Urbanism Department, Delft University of Technology, Delft, The Netherlands

**Keywords:** climate change, mitigation, adaptation, built environment, coastal cities

## Abstract

Gdansk in Poland and the Netherlands share a long-term relationship that started with the establishment of Dutch Mennonites in the Vistula delta in the 16 ^th^ Century. Climate-change figures show that both the Polish Gdansk and DutchRotterdam deltas will suffer flooding due to sea level rise, with accumulating severe rainfall accompanied by high river levels; reasons that led to a comparison of the adaptation measures taken. On the basis of the crossover comparison studied, it can be concluded that Poland and the Netherlands have a virtually identical approach when it comes to climate-change impacts on their current situation. With regard to the long-term climate-change trend, the Netherlands in exploring for the future more ‘anticipatory’ measures with the development of new scenarios for the protection of land and cities. In the Netherlands the use of Hackathon approach is thereby used more often to explore such scenarios. The interaction between the experts and stakeholders of different expertise in this methodology show to lead to creative and new perspectives. This approach may also be recommended for the situation in Gdansk.

## Northern EU Delta’s under climate-change pressure

Climate-change sea-level rise is increasingly seen as the major threat for coastal cities in Europe and globally, as most cities are historically situated in coastal delta’s ( Angel *et al.*, 2012). Despite the fact that the climate-related threats are diverse and accumulation determines the real impact, the sea level rise for many of these cities makes an unexpectedly large contribution to this; being an important insight of and foundation for the EU H2020 ‘ *SOS Climate Waterfront’* project ( SOSCW, 2019). Within Europa, the focus of this EU project, the situation in the Northern area, the area of the North Sea and the Baltic Sea, is more complex due to zones with land rise and land decline. Secondly, some areas lay under sea-level since centuries and have a high risk of severe flooding in combination with water impoundment by north-western storms [due to the fact that both seas have a funnel shape]. A situation that is becoming more critical due to the climate-change sea-level rise trend ( EEA, 2021).

**Figure 1.**
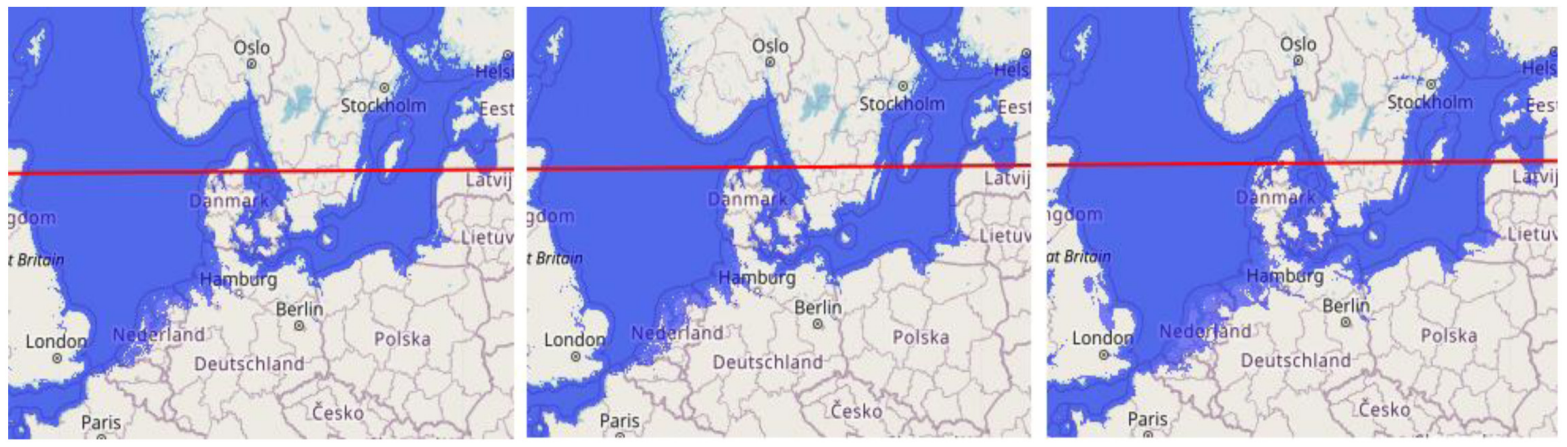
The land flooding of the northern zone of Europe, at 1-, 5- and 10-meters sea level rise from left to right (pictures made by using www.floodmap.net an open-access governmental website); with an indication of the zones of land rise [above the red line] and decline [below the red line] [1 cm = 400 km].

In Figure 1 in the three pictures is shown how land of the surrounding countries will theoretically become flooded by sea-level rise, where the effect of land rise has not been processed yet; see the zones of land rise and decline on Figure 1. The situation of Scandinavia however to be taken into account, is different from the rest of Europe. Here the land is still uplifting due to the two-kilometre-thick ice layer pressing the land down, that left the area a 10.000 years ago ( NLS, 2021). The rise of the land of Scandinavia, the fastest in the centre in the Baltic Sea in between Finland and Sweden, is 0,01 meter yearly, faster than the actual and predicted sea-level rise due to climate-change [chapter 2]. Hugo Sanders, participant in this EU H2020 project, observed himself during his 2020 visit to Finland that the village of Kalajoki on the west coast of Finland has already been moved to the sea several times because the harbour became too shallow for the ships. How different to the zone south of Scandinavia where the land declines due to peat oxidation and clinching of low-lying clay layers.

**Figure 2.**
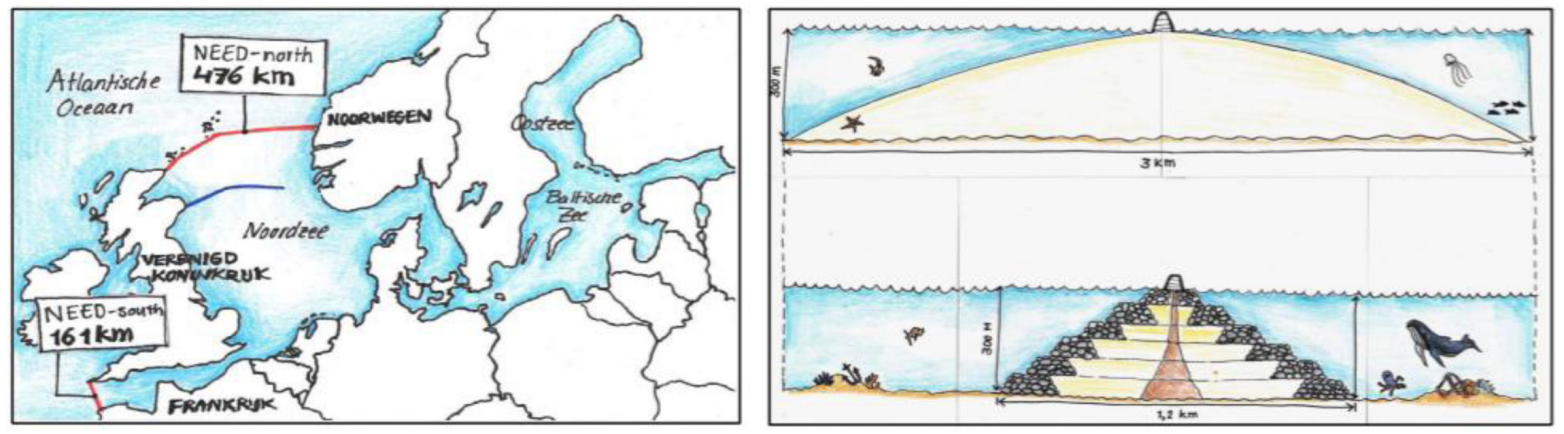
Impressions of ‘Damming the North Sea’ (source: TU Delft Delta magazine 2021).

With the recent IPCC report as reference, it’s not only this zone south of Scandinavia that due to land inclination will suffer more from the predicted sea level rise, this is a global problem to handle, with short and long-term adaptation measures to develop. About the Netherlands it can be said, that there are decades of experience in adaptation measures; strategies that are copied into the world. An example recently is the proposal for the area north and south of this zone-boundary, as given by a Dutch-Swedish scientist combination; the plan to close off the North Sea and thus the Baltic Sea, with a dam from the Atlantic Ocean ( Groeskamp & Kjellsson, 2020), see Figure 2.

This proposal, however, in addition to the enormous costs and the difficult technical feasibility, only blocks sea-level rise and does not solve the accumulation of the other water-related threats like high river levels, severe rainfall and storm surge from the sea, accompanied by high waves. That’s why, to the opinion of the Dutch team in the ‘SOS Climate Waterfront’ project; more adaptation solutions for delta areas should be explored, which provide security for the short and the long term. Not only because of the economic importance of coastal harbours and their hinterland, also for prevention of the historic inner-cities that are so vulnerable for higher water-levels.

With the focus on adaptation measures, because of the short-term risks for cities and their inhabitants; the initiative is taken to compare the Gdansk and Rotterdam delta situation, the two main harbours in this Baltic Sea and North Sea area. The idea is, that comparing the local and national taken and explored measures, should be educational for both cities, their countries and other coastal harbour delta cities. Within memory that the Gdansk delta with its surrounding polders, was created by the Dutch in the 16 ^th^ century, does this research fit in well with this historical tradition. This approach also fits the goals of the ‘ *SOS Climate Waterfront’* project, to explore robust climate-change water-related action-perspectives for the benefit of other coastal cities in Europe.

What sets as the research-question: *‘What lessons can be learned from comparing the Gdansk and Rotterdam Delta’s water related climate-change threats and adaptation measures that are executed and in planning, and what action perspectives can be recognized therein, from which these two cities and possibly also other coastal cities can benefit.’*

For answering this double question, Hugo Sanders from the Dutch project team visited Gdansk in 2019 to discuss the situation on its location. Afterwards three researchers from the Dutch team including himself, worked out the Gdansk and Rotterdam situation in the Netherlands [chapters 2 and 3], followed by crossover comparison (focusing on the factors that can be mutually compared) for exploring action perspectives, as a result of presentations and discussion during the 2019 ‘SOS Climate Waterfront’ seminar in Gdansk with additional desktop searching [chapter 4] with conclusions and acknowledgements [chapter 5]. For which a conceptual model is chosen and worked-out [chapter 4].

The research approach thereby follows the set-up of other comparison studies, from which most compare harbour situations among continents. For instance, the comparison of the Rotterdam-Guangzhou China situation [the Pearl River study project of Urbanism, Delft University of Technology] and Hamburg-Rotterdam ( Huang-Lachmann & Lovett, 2016).

## The Gdansk Baltic-sea climate-change delta situation

The Baltic Sea borders nine countries, is 1,600 km long, 193 km wide at its maximum and only 55 meters deep on average. The climate differences are huge in this area; strong long winters in the north and a mild continental climate in the south. This affects the water conditions and therewith the coastal circumstances and its water-related threats. Climate change influences this situation due to sea level rises, winter sea level changes, changing pole-tides, wind-induced water backlash, and the increasing water level changes in the sea joining rivers ( Ekman, 2009). The interaction of these influencing factors is complex, and the water level and the fluctuations therein are particularly location dependent ( Omstedt *et al.*, 2004). These factors have changed more drastically over recent years due to climate-change ( BACC Author Team, 2008).

**Figure 3.**
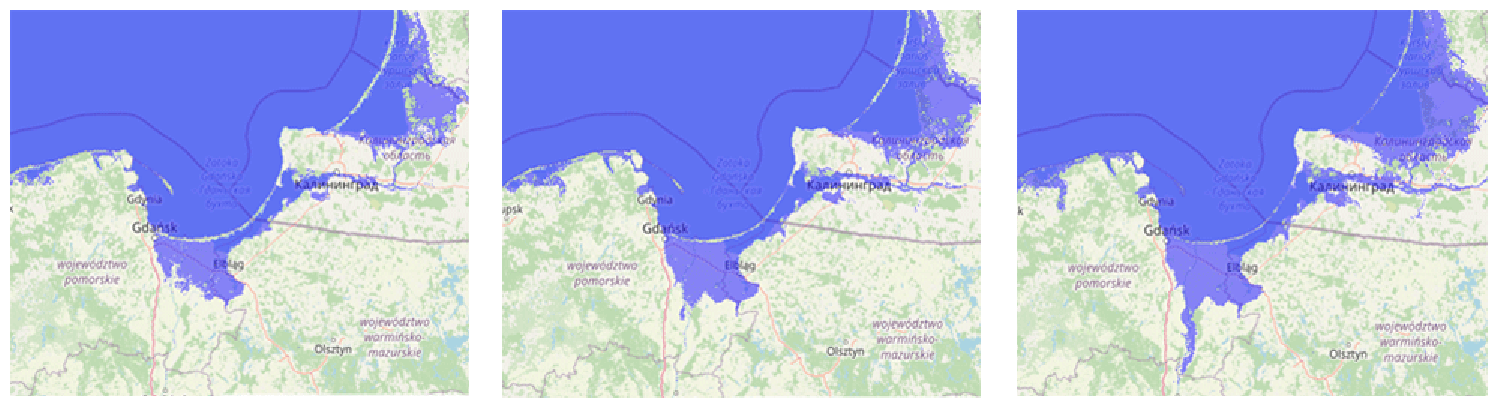
The land flooding at 1-, 5- and 10-meters sea level rise from left to right (pictures made by using www.floodmap.net, an open-access governmental website, with OpenStreetMap © OpenStreetMap contributors) [1 cm = 50 km].

The situation of the Scandinavian land north of the Baltic Sea is quite extraordinary. Here the land still rises every year after the former ice ages. The weight of the melted ice sheets is taken away and the land is returning to its former position. This is quite different from the southern Baltic Sea shore where the land is subject to subsidence ( Flemming *et al.*, 2017). There are many developing factors that together predict that the coasts around the Baltic Sea will change remarkably due to climate change in the years to come ( Labuz, 2015). According to the EC Inventory ( EEA, 2006), the Polish coastal zone is highly vulnerable to climate change, although relatively few people live along this 634 km coastline. See Figure 3 for an impression of the influence on the Polish coastline in case of rising sea-level rise.

Gdansk is situated along the Baltic Sea coast in the Gulf of Gdansk, west of the great Zulawy polder area, also called the Vistula delta because of the river Vistula that dominates the area. The area and the landscape are protected because the Vistula delta is a nature reserve, is responsible for 6% of the Polish agricultural food production and is an important source of drinking water. Handling the climate-change water-related impact on this delta, is especially urgent because besides sea-level rise and land inclination, there is an increasing accumulation of high river water levels because of severe rainfall in the last decade, especially coming from the higher hinterland. For the city of Gdansk, the situation has become more urgent in recent years as the city border lies at the eastern funnel-shaped outlet of the Baltic Sea with an open

The area of the city of Gdansk is characterised by an anthropogenic landscape of polders, dried marshlands and former oxbow lakes that have suffered periodic flooding during the last few centuries. The situation changed with the construction of the bypass channel of the Vistula river east of Gdansk city in 1895, leading directly to the Gulf of Gdansk ( WMO, 2005). However, climate change caused new flooding by cumulating sources in 1924 (huge snowmelt and rainfall), 1955 (strong storm), 1956 (ice-jam), 1983 (accident), and 2001 (torrential rainfall) ( Cyberski *et al.*, 2006). The coastal situation of Gdansk situated within the Gulf of Gdansk is thus determined by several climate change factors whereby the configuration of the Baltic Sea as a whole plays a role. The main influencing factors are: the higher seawater temperature; decreasing ice formation; increasing water import from rivers with their fluctuations along the Baltic Sea coast and in specific locations; increasing water backlog due to storm surges and more heavy clustered rainfall; the rising and falling of the land, which differs from the southern coast on the northern coast, whereby the effects differ locally along the 1,900 km long coast ( Ekman, 2009). For which computer simulations are under development at institutes in Poland https://www.climatechangepost.com/poland/climate-change/ ( Climate-change Post, update 2021) and at NIOZ in the Netherlands as well.

The current situation is that Gdansk city and the eastern polder area remains dry by constant action: dike reinforcement and drainage pumping the water out of the delta by channels and the rivers to the sea. For the city of Gdansk, the planned climate change measures are: 1) long term coastal protection strategies; 2) flood warning system for the safety of residents; 3) canaling of the Vistula river to the Gulf of Gdansk in 1840 and 1895; 4) reservoirs for flood protection built on streams in cascades; 5) expansion of the city drainage system for capturing heavy rainfall; 6) creating a new. Lower risk riverfront and city architecture. Measures are taken to decrease the impact on buildings in the event of flooding, for example placing crucial functions on the first floor higher, and creating parks and gardens that allow rainwater to pass into the absorbing capacity of the soil ( ModE, 2010; ModE, 2019).

An additional factor is, how the impact of climate-change in the other corners of the Baltic Sea influence the situation in Gdansk and its surroundings. For instance; the reduction in ice-formation in the northern part of the Baltic Sea will in wintertime reduce the rise in seawater level on the southern coast ( Ekman, 2009). Another factor would be; that unless the costs of extra measures at this south coast in general will be less than the loss of value in the coastal area of Poland, the total investment will probably and actually be too much for the region ( Zeidler, 2015).

Results of the discussion about this situation in Gdansk within the ‘SOS Climate Waterfront’ project on location and within the Dutch team, yielded: 1) learning approaches from the probably similar situations in the Gdansk eastern funnel-area of the Baltic Sea could deliver new measuring insights, by studying the coastal; situation of Estonia, Latvia, Lithuania and Finland; 2) studying the relationship of land-rise at the northside of the Baltic Sea, for instance the situation of nearby Finland, is necessary to know the southern situation better; and 3) measures taken at the coast of Stockholm, a for Gdansk apparently other situation at the Baltic Sea, could provide new opportunities. Additionally, 4) influences of climate-change on nature and its biodiversity should get attention, with as example: deforestation and destruction of river-vegetation accelerates the discharge of water from rivers.

## The Rotterdam North Sea climate change delta situation

The last severe flood in the Netherlands, called ‘Watersnoodramp’, happened in February 1953 whereby 165 hectares of land flooded, mainly in the southwest of the country. In total, 1836 people lost their lives, 10k people lost their homes from the 72k that had to be evacuated, and approximately 50k cows and 150k chickens died. This unexpected disaster became the start of ‘Deltaplan’ to protect the Netherlands from the sea in the future. With the installation of the governmental ‘Delta Commission’ in 1953 two months later, plans to shorten the coast with 700 km of dikes started ( Deltawet, 1958). During the years after, the safety of the important city of Rotterdam was ensured by this program through construction of the ‘Maesland barrier’: the construction of two moving arms that turn into the Rhein-river waterway at high tide to close it off. In 2011 this ‘Deltaplan’ changed drastically; due to climate-change. The Delta Commission was directed to make plans to address seawater level rises and other climate-change coupled impacts in the future ( Deltawet, 2011).

**Figure 4.**
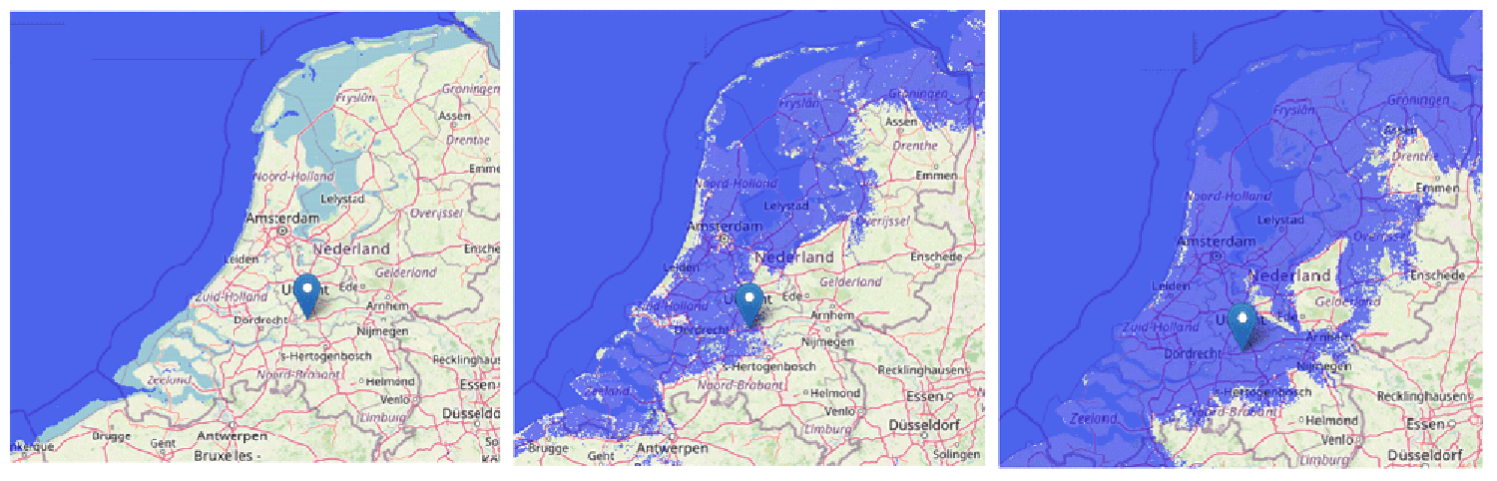
The situation of the Netherlands in relation to sea level: actual situation behind the dikes (left), flooding in case of 1.0 meter higher sea level without dikes (middle), and in case of 10.0 meter (right) (pictures made by using www.floodmap.net, an open-access governmental website, with OpenStreetMap © OpenStreetMap contributors) [1cm = 50 km].

Based on the recent Delta-Commission statement, the Dutch coast system can handle 10 meters of sea level rise totally, assuming there are no budget constraints. Flood scenarios show that 50% of the country will be flooded in that extreme situation when there would be no coastal dikes ( Haasnoot *et al.*, 2020), which concerns the part of the country where almost 80% of the country population lives and where the cities Amsterdam and Rotterdam, the most important economic motors of the country, see Figure 4.

The most recent advice from this commission of 15 September 2020 ( Deltaprogramma, 2021) focussed on: 1) water security, 2) freshwater availability; and 3) spatial design, all related to climate-change impact. The Delta Commission thereby concluded that the Dutch coastal defence system can handle the 100 cm sea level rise that may happen at the earliest in 2100 according to worse-case scenarios based on the 2014 Fifth assessment IPCC institute report. However, the newest 2021 Sixth IPCC report states that this worse-case scenario could be more serious. That’s why with more urgency in the Netherlands for the Rhein delta adaptation measures are in development and construction. Measures in and around the city of Rotterdam to be taken, following statements of this Delta Commission, are: 1. coastal and hinterland dikes have to be reinforced, 2. rivers need to be upstream widened for water collection capacity at high river levels, 3. buildings and housing complexes along river embankments must be redesigned so that high river levels, as a result of the combination of high-water discharge from the rivers and high sea levels, cannot cause severe damage in the coming centuries, and 4. rain collection reservoirs are being built in the city.

Most worrisome for an update of the 2011 Deltaplan will be the major operation to gradually strengthen and raise all dikes, the primary sea defence and the polder dikes being the second and third defence system of the Dutch country. The central factor behind the risk analysis of the primary sea defence is the accumulation of the rising sea-level, temporary high river levels, and storm with high swell waves. Additionally drier periods can weaken these defence system dykes( Deltawet, 2011).

These effects also have secondary impact on the overload of sewer systems in cities, the subsidence of old houses, and the fertility of agricultural land for which salinization is an important factor. The production of Dutch tulip bulbs and potatoes already suffer from low salinization. The most drastic impact on national scale, however, is the expected future flooding of the large cities in the western part of the Netherlands and the stagnation of economic growth in the adjacent industrial sites, which a major impact on national scale.

**Figure 5.**
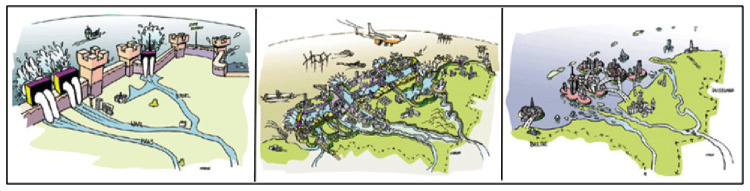
Three scenarios for handling extreme sea level rise in the Netherlands; the three scenarios from left to right. (Copied from the Hackathon report with permission of Deltares) ( Haasnoot *et al.*, 2019).

To stay ahead of this development, more drastic measures in the coming centuries will be needed according to the 2020 Delta Commissioner advise to address the impact of water-related climate-change on the long-term. In 2019 therefor a ‘Policy Hackathon’ was organized ( Haasnoot *et al.*, 2019) to explore robust scenarios and solutions for the long run. This resulted in the three main scenarios for handling extreme sea level rise in the Netherlands: 1. defending the delta by fortification using a high dike around the country; 2. defending the delta by creating a new barrier off the coast with the advantage of new land for housing and tourism; 3. defending the most important economic areas including the cities using dikes with the sea coming into the land, see Figure 5.

Comments on the three Hackathon scenarios as discussed within the ‘SOS Climate Waterfront’ project Dutch team: 1. the choice to raise the outer dikes has limitations, because the subsoil has limited bearing capacity, the drainage of river water is hindered and because the land behind the dike will experience problems in draining rainwater to the sea, 2. the construction of a second defense zone outside the primary sea defense not only creates extra land and recreational facilities that could contribute to funding the defense zone, but also a transition zone is created by which the river water could flow easier to the sea and the land behind the dike is protected in a multi-value and multi-usable way, and 3. giving back land to the sea does not fit into the Dutch tradition and it breaks down the many investments made in the past, it is also practically difficult to keep the large cities such as Rotterdam in the form of islands alive, because they are seaports with industry and logistics handling. The fact that the Netherlands is still an important agricultural country on a global scale is thus negated.

## Crossover comparison and action perspectives

When comparing the climate-change adaptation measures of the cities Gdansk in Poland and Rotterdam in the Netherlands, both are situated in the heart of the country’s most important delta and both locations are similarly in relation to the sea and to the hinterland. Both cities: 1) are located by an enclosed sea in the northern hemisphere, in a delta with surrounding ‘polder’ areas; 2) face sea level rise; 3) experience backwater during storms; and 4) are located on a river in the delta where the river level fluctuates strongly, caused by meltwater upstream, heavy rainfall alternated with dry periods, with peaks occurring when these influences coincide. The situations are so similar that it is interesting to learn from the mutual experiences in the field of climate-change interventions.

It is valuable to compare why and to what extent is chosen for short or long-term adaptive measures, in what capacity and proportions. Because, as concluded based on the Dutch situation [chapter 1], long-term adaptive measures are meant to be designed to change with changing climate conditions and theoretically could be more flexible and. durable.

**Figure 6.**
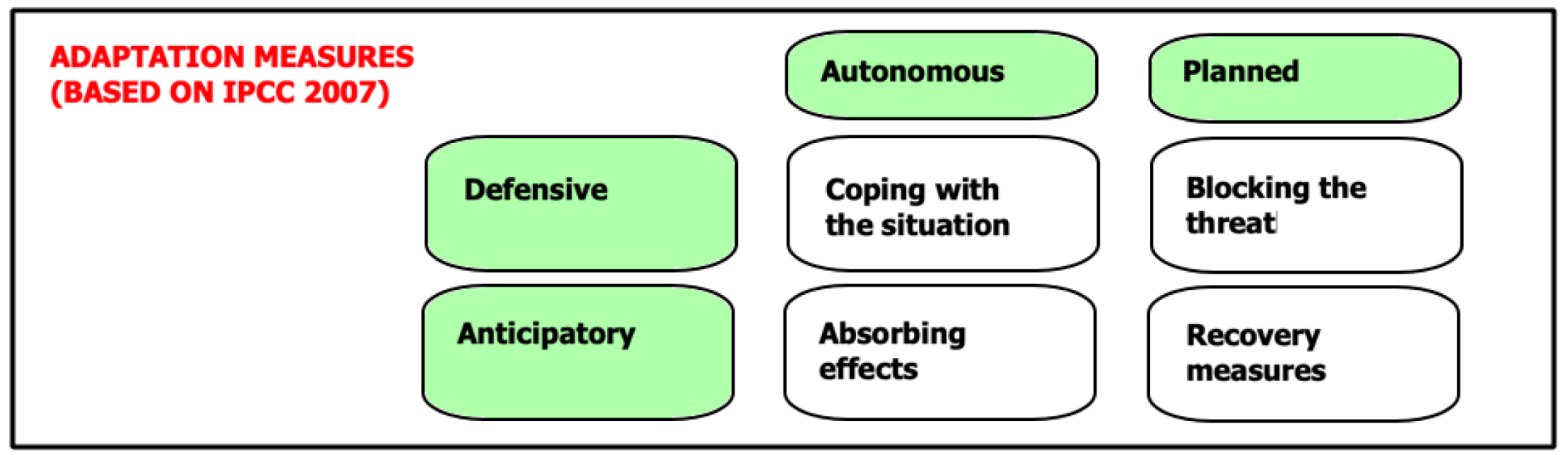
Diagram for climate-change adaptation measures (Adapted from IPCC 2007 Report, with permission of Cambridge University Press, 20 January 2021) ( IPCC, 2007).

To explain this opposite type of adaptation measures in comparison, the ‘Astra’ model (based on IPCC models) that places categories of adaptation measures in a diagram is used for comparison and analyses ( Hilpert *et al.*, 2007). distinguishes between ‘Autonomous’ (short-term) and ‘Planned’ (long-term) measures dividing these into ‘Defensive’ and ‘Anticipatory’ measures, see Figure 6.

Using the different measures realized and planned for the cities of Gdansk and Rotterdam are categorized besides each other, see Table 1. This table is created for crossover comparison of the measures mentioned [chapter 2 and 3]; focusing on the factors that can be compared for benefit of both city locations, for working-out action perspectives, for these cities and possibly also for other cities. 

**Table 1.** Inventory and comparison Gdansk and Rotterdam climate-change adaption measures.

MEASURES	Gdansk	Rotterdam
**Coping the situation**	• New riverfront and city architecture	• New riverfront and city architecture.
**Absorbing effects**	-	• Long-term measures: scenarios 1 and 3
**Blocking the threat**	• Plan coastal protection strategy • Flood warning system residents. • City drainage system for heavy rainfall • Reservoirs for flood in rivers • The ‘Vistula’ canals to ‘Gdansk Gulf’	• Raising primary and inner dikes. • Closing rivers in case of emergency. • Rain collecting reservoirs. • Widening upstream river beds.
**Recovery measures**	-	• Long-term measures: scenario 2.

The location conditions of Gdansk and Rotterdam are quite similar, the differences are the size and logistics position in Europe. Both cities and their countries are aware of their vulnerability to climate-change impacts because of their delta situated locations; which they show by working-out measures and implement them.

A similarity in the measures that have been realized as the influx of water away from the cities is slowed down by widening rivers, rainwater reservoirs are constructed and dikes are raised, is that the primarily focus is on ‘defensive’ measures. Thus, avoiding flooding and the accumulation of effects as much as possible. The difference in general is that for the city of Rotterdam more ‘anticipatory’ adaptation measures are in development, largely fulfilled by measures initiated at national level.

The fact that more effort is in progress creating ‘anticipatory’ short and long-term adaptation measures for the situation of Rotterdam, instigated from the national level, must lay in the tradition of the Netherlands to protect the country against high water, and the financial situation may also play a role in this. Nevertheless, the ‘defensive’ flood protection measures taken at Gdansk and Rotterdam is not that different, given the difficulty of the ‘anticipatory’ measures being worked on.

## Conclusions and acknowledgements

It should be noted that it is apparently difficult for both locations to find ‘anticipatory’ adaptation measures. While it is precisely in these measures to find action-perspectives, to create measures of a more structural nature. A good example of such coastal climate-change measures is the so-called ‘Sand Motor’ created at the northwest coast of the Netherlands ( Climate Adapt, 2021): erosion of the dunes is prevented by creating a sand depot against the dunes in the coastal current, so that the erosion caused by storms is compensated in a natural way. However, it concerns a sparsely populated area where the space is not needed for industry and shipping. The problem of small measures for industrial cities such as Gdansk and Rotterdam are apparently that the required space is already in use. Still, it is promising that the Hackathon method offers a creative and promising solution, by involving all stakeholders around the problem. With crosspollination of knowledge and perspectives, adaptation measures can be explored in a more active and societal acceptable way. There way the action-perspective found in the Hackathon methodology is therefor process-based and not an actual solution scenario, but is being used more and more for climate-change issues and situation [also called Climathon]. Examples are: the 2020 Bangkok Climate Change Hackathon, the 2020 Open-source Presidential Hackathon Taiwan and the virtual EU Climathon program [ https://climathon.climate-kic.org]. The Hackathon research methodology that first started with private group sessions, shows with these examples to be developed in an open research methodology in which researchers and problem owners worldwide can participate. This development as it is going makes this an interesting methodology to use for climate-change challenges in a multitude of situations.

## Data availability

All data underlying the results are available as part of the article and no additional source data are required.
