# Supplementary material for: Crossover comparison of climate-change adaptation measures taken in the Gdansk (Baltic-sea) and Rotterdam (Nord-sea) deltas
Source: Open Res Eur. 2021 Oct 13;1:9. Originally published 2021 Mar 24. [Version 2] doi: 10.12688/openreseurope.13125.2 (PMC10445832; doi:10.12688/openreseurope.13125.2)
Supplement: Peer review history

# Peer review: Reviewer response for version 2

**DOI:** 10.21956/openreseurope.15302.r27797

**Published:** 2021-10-25

**Authors:** Tineke van der Schoor

**Affiliations:** 1 Research Centre for Built Environment NoorderRuimte, Hanze University of Applied Sciences, Groningen, The Netherlands

The author's response lists several changes that have been made, navigation would be easier for the reader if the chapters/ sections were numbered.

 The Hackathon method is now briefly described, and the reader is referred to another report for more information - however, this is in Dutch. Furthermore, a methodology article on Hackathon or Climathon as a research method would be very interesting.

Is the study design appropriate and does the work have academic merit?

Yes

Is the work clearly and accurately presented and does it cite the current literature?

Yes

If applicable, is the statistical analysis and its interpretation appropriate?

Not applicable

Are all the source data underlying the results available to ensure full reproducibility?

Yes

Are the conclusions drawn adequately supported by the results?

Yes

Are sufficient details of methods and analysis provided to allow replication by others?

Partly

Reviewer Expertise:

NA

I confirm that I have read this submission and believe that I have an appropriate level of expertise to confirm that it is of an acceptable scientific standard.

# Peer review: Reviewer response for version 2

**DOI:** 10.21956/openreseurope.15302.r27798

**Published:** 2021-10-20

**Authors:** Leslie Mabon

**Affiliations:** 1 Scottish Association for Marine Science, University of the Highlands and Islands, Inverness, UK

This is a much improved text, thanks very much for the opportunity to review it. Two very minor comments that need be addressed:

1. The conclusion would benefit from a couple of sentences that reiterate the key findings of the research, in addition to the material that is there already.
2. The article needs thorough proofreading as there are several spelling and typographical errors (e.g. the title of the first section does not need an apostrophe in Deltas).

Is the study design appropriate and does the work have academic merit?

Partly

Is the work clearly and accurately presented and does it cite the current literature?

Partly

If applicable, is the statistical analysis and its interpretation appropriate?

Not applicable

Are all the source data underlying the results available to ensure full reproducibility?

Partly

Are the conclusions drawn adequately supported by the results?

Partly

Are sufficient details of methods and analysis provided to allow replication by others?

Partly

Reviewer Expertise:

climate change adaptation; environmental governance; environmental policy

I confirm that I have read this submission and believe that I have an appropriate level of expertise to confirm that it is of an acceptable scientific standard.

# Peer review: Reviewer response for version 2

**DOI:** 10.21956/openreseurope.15302.r27796

**Published:** 2021-10-14

**Authors:** Michael Nones

**Affiliations:** 1 Institute of Geophysics, Polish Academy of Sciences, Warsaw, Poland

Dear Authors,

 Many thanks for having revised the article following my comments.

 In my opinion, the present version is more scientifically sound and could help in managing delta cities exposed to climate change.

Is the study design appropriate and does the work have academic merit?

Yes

Is the work clearly and accurately presented and does it cite the current literature?

Partly

If applicable, is the statistical analysis and its interpretation appropriate?

Not applicable

Are all the source data underlying the results available to ensure full reproducibility?

Yes

Are the conclusions drawn adequately supported by the results?

Yes

Are sufficient details of methods and analysis provided to allow replication by others?

No

Reviewer Expertise:

River morphodynamics.

I confirm that I have read this submission and believe that I have an appropriate level of expertise to confirm that it is of an acceptable scientific standard.

# Peer review: Reviewer response for version 1

**DOI:** 10.21956/openreseurope.14195.r26990

**Published:** 2021-06-22

**Authors:** Leslie Mabon

**Affiliations:** 1 Scottish Association for Marine Science, University of the Highlands and Islands, Inverness, UK

Thank you for the opportunity to review this work. The option the portal gives me for recommendation is titled 'Not Approved', which sounds a lot more harsh than I intend my recommendation to be! If this was a more traditional academic journal, I would probably select 'Major Revisions'. I believe that this can become a scientifically sound piece of research with a bit of expansion and re-writing as follows. So please do not be put off by the 'Not Approved' recommendation.

 This is an interesting and valuable paper which compares approaches to flooding in two delta areas in the northern part of Europe – Gdansk and Rotterdam. Approaches to dealing with flooding under conditions of rising sea levels and increasingly frequent extreme events are fast becoming an area of scholarly concern, so this is a timely contribution from a part of the world that has not received as much scholarly attention as other low-lying areas to date.

 However, whilst the premise of the paper is valuable and I strongly believe there is important data in here, I believe the paper needs some reworking and development to really bring out the scientific insights. As it stands this is fine as a case report, however if it is to be developed into a scholarly contribution (either here or for publication in another venue) then more engagement with the existing research literature is needed. My suggestions for how to do this are as follows.

1. You should make much more of the comparison between the two cases. At present, there is a useful table on the adaptive activities of each city, and a few paragraphs on some of the similarities and differences between the cases. For an international readership, the comparison between the cases, and especially what we can learn from where there are differences and why, is going to be equally as valuable as the descriptive information on what is happening in each of the cities. I would therefore suggest you to significantly expand the comparison section and to consider how your findings build on what is already discussed in the literature. Looking at how the cities might learn from each other could be an especially valuable line of enquiry, given the growing scholarly interest in how cities can learn from each other (both formally and informally) to develop resilience. See e.g.
  Acuto, M., & Leffel, B. (2020). Understanding the global ecosystem of city networks. Urban Studies, 004209802092926. https://doi.org/10.1177/0042098020929261
  Bai, X., Nagendra, H., Shi, P., & Liu, H. (2020). Cities: build networks and share plans to emerge stronger from COVID-19. Nature, 584(7822), 517–520. https://doi.org/10.1038/d41586-020-02459-2
2. I was especially interested to read about the Policy Hackathon approaches to developing flood scenarios and solutions in Rotterdam. I wonder if this is something you could look into more in the discussion, perhaps as a response to my point above? For instance, what new and different ways are cities using to engage a range of knowledges and expertises in the governance of challenging and uncertain issues such as flooding, and how can these Hackathon-type approaches produce more robust outcomes compared to more traditional approaches to science-policy-practice interaction? (see for example the Presidential Hackathon organised in Taiwan in 2020, which used open data to encourage teams to develop solutions for each of the Sustainable Development Goals).
3. If the text here is going to stand as a scientific contribution, it would be helpful to add an introductory section which sets out the context of the work and the key scholarly (or even policy) issues that the work speaks to. For example, a lot of work has been done on adaptation in deltas in lower-latitude country contexts, however there is perhaps not much on adaptation in deltas at higher-latitudes. Nonetheless, the last year or so has seen a huge upswing in research into adaptation for coastal areas in higher-latitude countries (see e.g. this recent special issue https://link.springer.com/article/10.1007/s13412-021-00700-6) so there is an emerging science-policy requirement that your work comes at just the right time to engage with.
4. Methodology. Again reflecting the above, if the work is going to be developed it would be worthwhile having a more explicit Method/Methodology section, as readers will want to see this. So for example, you mention you did a cross-over analysis. What is meant by a cross-over analysis, and how did you do this in practice? Take, for example, the very helpful Table 1 you have – where does this information come from (e.g. policy analysis, interviews?) and how was it sourced/analysed? How can you be sure you got all the relevant information for each city, and how can you demonstrate to the readers that you did the analysis in a rigorous way? Reference to some standard methods textbooks and/or other papers which use a similar methodological approach will help to show the rigour of the work.

Is the study design appropriate and does the work have academic merit?

Partly

Is the work clearly and accurately presented and does it cite the current literature?

Partly

If applicable, is the statistical analysis and its interpretation appropriate?

Not applicable

Are all the source data underlying the results available to ensure full reproducibility?

Partly

Are the conclusions drawn adequately supported by the results?

Partly

Are sufficient details of methods and analysis provided to allow replication by others?

Partly

Reviewer Expertise:

climate change adaptation; environmental governance; environmental policy

I confirm that I have read this submission and believe that I have an appropriate level of expertise to state that I do not consider it to be of an acceptable scientific standard, for reasons outlined above.

## References

[1] : Understanding the global ecosystem of city networks. *Urban Studies* .2021;58(9) : 10.1177/0042098020929261 1758-1774 10.1177/0042098020929261

[2] : Cities: build networks and share plans to emerge stronger from COVID-19. *Nature* .584(7822) : 10.1038/d41586-020-02459-2 517-520 10.1038/d41586-020-02459-2 32843732

[3] : Introduction: Managed retreat and environmental justice in a changing climate. *Journal of Environmental Studies and Sciences* .2021; 10.1007/s13412-021-00700-6 10.1007/s13412-021-00700-6

# Response

**Published:** 2021-10-06

**Authors:** Fred Sanders

**Affiliations:** CPONH NGO, The Netherlands

Dear 3rd Reviewer Leslie Mabon, we worked-out your comments in our revised paper, according to your three most notable comments:

1. We added a new chapter (1) to introduce the research question and the research methodology, and we split the analysis chapter in analysis (4) and conclusions (5) to make the comparison of both cities more clear, as you suggested. The research as we did has taken place within the confines of the EU H2020 'SOS Climate Waterfront' project and therefore does not extend to other comparable places. We explained that in the paper now. We added in the paper though parallel studies in and outside the EU, because its true the research in deltas gets growing attention.
2. The conceptual model for the analysis we have worked-out further and the presentation of the comparison is given attention, to make the differences of the comparison more clear.
3. Examples of the use of the Hackathon methodology are added, to make clear the growing potential of this approach, as you suggested. We think all your suggestions, the small ones and the more notable ones, all have helped by working-out our paper better. Your remarks stimulated us by addressing that comparisons like we did will help other coastal deltas to consider climate-change adaptation measures better.

Thank you for reflecting our work.

# Peer review: Reviewer response for version 1

**DOI:** 10.21956/openreseurope.14195.r27000

**Published:** 2021-06-01

**Authors:** Michael Nones

**Affiliations:** 1 Institute of Geophysics, Polish Academy of Sciences, Warsaw, Poland

**General comment**: even if the study goals can be derived from the last section, I suggest adding a few more details at the very beginning of the article, to better pinpoint what is the scope of the research, and how your work combines with the existing literature. 

**Abstract: **please add the country of Gdansk (as done for Haarlem).

**Figure 1**: please add a graphical scale and the coordinates, to better place the study area. (same for Fig. 5)

**Figure 2**: what is the scope of presenting these photos? What is the added value of this figure? (same comments for Figs. 3 and 4)

**Language**: even if I am not a native English speaker, I would like to advise you that a double-checking of the language is needed

Is the study design appropriate and does the work have academic merit?

Yes

Is the work clearly and accurately presented and does it cite the current literature?

Partly

If applicable, is the statistical analysis and its interpretation appropriate?

Not applicable

Are all the source data underlying the results available to ensure full reproducibility?

Yes

Are the conclusions drawn adequately supported by the results?

Yes

Are sufficient details of methods and analysis provided to allow replication by others?

No

Reviewer Expertise:

River morphodynamics.

I confirm that I have read this submission and believe that I have an appropriate level of expertise to confirm that it is of an acceptable scientific standard, however I have significant reservations, as outlined above.

# Response

**Published:** 2021-10-06

**Authors:** Fred Sanders

**Affiliations:** CPONH NGO, The Netherlands

Dear 2nd Reviewer Michael Nones, we worked-out your comments in our revised paper, according to your three most notable comments:

1. We rewrote the abstract to mention more clearly that the comparison concerns the cities of Gdansk and Rotterdam and not Haarlem, we skipped Haarlem.
2. We added the graphical scale of the maps in text of the explanation at the concerning three figures.
3. We removed the pictures because these we not that essential. They came from the case-study presentation at the 2019 Gdansk conference, from which we worked-out further this paper for publishing.

Thank you for reflecting our work.

# Peer review: Reviewer response for version 1

**DOI:** 10.21956/openreseurope.14195.r26652

**Published:** 2021-04-28

**Authors:** Tineke van der Schoor

**Affiliations:** 1 Research Centre for Built Environment NoorderRuimte, Hanze University of Applied Sciences, Groningen, The Netherlands

This article compares the approaches of the cities Gdansk and Rotterdam to the protection for future consequences of climate change. The authors argue that there are many similarities between the situation of these two cities, and that they could profit from exchanging knowledge and experiences. The article starts out with a description of the local and regional situation of the two cities in their surrounding environment, the current water management measures as well as the planned climate change measures for long term protection. For the situation in Poland, much can be learned from comparisons with other Baltic sea cities and regions. On the other hand, the situation in the Netherlands is seen as more isolated. A few remarks on this article are the following. Firstly, the method and research design should be included in the article. What research methods are used, what is cross-over analysis and how is it performed. Secondly, the section on analysis and conclusions is rather short. It could be useful to divide this in two sections and take more room to compare the different approaches. For example, the monetary and ecological costs of approaches are only briefly mentioned, these could be further discussed. Third, the situation in the Netherlands is not as isolated as argued, for example the new ‘Deltaplan’ would entail cooperation with all countries bordering the North Sea. Furthermore, there are comparable situations in other North Sea countries, for example in Germany (Hamburg) and Denmark. Such examples from farther afield could be added to or referred to in the article.

Is the study design appropriate and does the work have academic merit?

Yes

Is the work clearly and accurately presented and does it cite the current literature?

Yes

If applicable, is the statistical analysis and its interpretation appropriate?

Not applicable

Are all the source data underlying the results available to ensure full reproducibility?

Yes

Are the conclusions drawn adequately supported by the results?

Yes

Are sufficient details of methods and analysis provided to allow replication by others?

Partly

Reviewer Expertise:

NA

I confirm that I have read this submission and believe that I have an appropriate level of expertise to confirm that it is of an acceptable scientific standard, however I have significant reservations, as outlined above.

# Response

**Published:** 2021-10-06

**Authors:** Fred Sanders

**Affiliations:** CPONH NGO, The Netherlands

Dear 1st Reviewer Tineke van de Schoor, we worked-out your comments in our revised paper, according yo your three most notable comments:

1. We added a new chapter (1) to introduce the research question and the research methodology.
2. We divided the chapter with conclusions in the two chapters (4) and (5) analysis and conclusions to give, as you advised, that analysis more body and explanation.
3. We explained why we focused on the northern EU deltas at Gdansk and Rotterdam in the new introduction chapter (10) and gave some examples of research on other places at the Baltic Sea and in the world, to better position the research as you suggested.

Thank you for reflecting our work.
